# Successful Control of Soil-Transmitted Helminthiasis in School Age Children in Burkina Faso and an Example of Community-Based Assessment via Lymphatic Filariasis Transmission Assessment Survey

**DOI:** 10.1371/journal.pntd.0004707

**Published:** 2016-05-10

**Authors:** François Drabo, Hamado Ouedraogo, Roland Bougma, Clarisse Bougouma, Issouf Bamba, Dramane Zongo, Mohamed Bagayan, Laura Barrett, Fanny Yago-Wienne, Stephanie Palmer, Brian Chu, Emily Toubali, Yaobi Zhang

**Affiliations:** 1 Programme National de Lutte contre les Maladies Tropicales Négligées, Direction de la Lutte contre la Maladies, Ministère de la Santé, Ouagadougou, Burkina Faso; 2 Helen Keller International, Ouagadougou, Burkina Faso; 3 Health Sciences Research Institute (IRSS), Ouagadougou, Burkina Faso; 4 University of Ouagadougou, Ouagadougou, Burkina Faso; 5 Helen Keller International, New York, New York, United States of America; 6 Neglected Tropical Diseases Support Center, Task Force for Global Health, Decatur, Georgia, United States of America; 7 Helen Keller International, Regional Office for Africa, Dakar, Senegal; Emory University, UNITED STATES

## Abstract

**Background:**

Burkina Faso is endemic with soil-transmitted helminth infections. Over a decade of preventive chemotherapy has been implemented through annual lymphatic filariasis (LF) mass drug administration (MDA) for population aged five years and over, biennial treatment of school age children with albendazole together with schistosomiasis MDA and biannual treatment of pre-school age children through Child Health Days. Assessments were conducted to evaluate the current situation and to determine the treatment strategy for the future.

**Methodology/Principal Findings:**

A cross-sectional assessment was conducted in 22 sentinel sites across the country in 2013. In total, 3,514 school age children (1,748 boys and 1,766 girls) were examined by the Kato-Katz method. Overall, soil-transmitted helminth prevalence was 1.3% (95% CI: 1.0–1.8%) in children examined. Hookworm was the main species detected, with prevalence of 1.2% (95% CI: 0.9–1.6%) and mean egg counts of 2.1 epg (95% CI: 0–4.2 epg). Among regions, the Centre Ouest region had the highest hookworm prevalence of 3.4% (95% CI: 1.9–6.1%) and mean egg counts of 14.9 epg (95% CI: 3.3–26.6 epg). A separate assessment was conducted in the Centre Nord region in 2014 using community-based cluster survey design during an LF transmission assessment survey (TAS). In this assessment, 351 children aged 6–7 years and 345 children aged 10–14 years were examined, with two cases (0.6% (95% CI: 0.2–2.1%)) and seven cases (2.0% (95% CI: 1.0–4.1%)) of hookworm infection was identified respectively. The results using both age groups categorized the region to be 2% to <10% in STH prevalence according to the pre-defined cut-off values.

**Conclusions/Significance:**

Through large-scale preventive chemotherapy, Burkina Faso has effectively controlled STH in school age children in the country. Research should be conducted on future strategies to consolidate the gain and to interrupt STH transmission in Burkina Faso. It is also demonstrated that LF TAS provides one feasible and efficient platform to assess the STH situation for post LF MDA decision making.

## Author Summary

Burkina Faso is a West African country endemic with soil-transmitted helminth infections (STH). Mass treatment with albendazole has been implemented for over a decade in the country through annual mass drug administration (MDA) for lymphatic filariasis for population aged five years and over, biennial treatment of school age children with albendazole together with schistosomiasis MDA, and biannual treatment of pre-school age children through Child Health Days. A sentinel site survey in 2013 showed that STH infection in Burkina Faso was very low at 1.3%. Hookworm was the main species detected, but infection was low and light. An assessment was also conducted with two age groups in Centre Nord region through lymphatic filariasis transmission assessment survey in 2014. The results confirmed the low level of infection with soil-transmitted helminths in the region. Through large-scale preventive chemotherapy, Burkina Faso has effectively controlled STH in school age children in the country. The future STH deworming strategy may focus on consolidating the gain and interrupting the STH transmission in the country.

## Introduction

Soil-transmitted helminthiasis (STH), one of the major neglected tropical diseases (NTDs), is caused by a group of nematodes, namely hookworms (*Ancylostoma duodenale* and *Necator americanus*), *Ascaris lumbricoides* and *Trichuris trchiura*. Chronic infection with these parasites can cause malnutrition, iron deficiency, anemia and impairment of physical and intellectual development in school age children [1–3]. The transmission of STH is linked to poverty and lack of health practice, and associated with low parental literacy rates, poor hygiene and sanitation, and lack of access to safe and clean water [4–7]. The disease is widely endemic in the developing countries in the world and causes the highest burden of the NTDs among the poorest populations [8, 9]. Worldwide, it is estimated that about two billion people in developing countries are infected with one or more species of helminths [10, 11], with approximately 300 million people suffering from severe morbidity resulting in 10,000–135,000 deaths annually [12]. STH infections are normally treated by a single dose of albendazole or mebendazole [13]. World Health Organization (WHO) recommends controlling morbidity caused by STH infections through preventive chemotherapy with anthelmintic drugs in pre-school age children, school age children, as well as adolescent girls, women of reproductive age and pregnant women (second and third trimester) [11, 14]. The current global objective is to attain regular treatment of 75% of pre-school age children and school age children in all endemic countries by the year 2020 [15].

Burkina Faso is a West African country that is divided into 13 health regions with 63 health districts. The country has three sub climate zones: north-Sudanese in the south, sub-Sahelian in the middle and Sahelian in the north, with an annual rainfall between 400 and 1,200 mm [16]. The country is known to be endemic with STH according to historic data, but published literatures on population-based surveys is scarce [17–20]. A 1984 survey in two villages in Kaya in Centre Nord region showed that hookworm (*N*. *americanus*) prevalence was 14.7% in Louda and 9.3% in Damesma, while prevalence of *A*. *lumbricoides* or *T*. *trichiura* was found to be below 0.5% [17]. During the baseline data collection for schistosomiasis and STH in 2004–05 in four regions (Boucle du Mouhoun, Nord, Sahel and Sud Ouest) highly endemic with schistosomiasis, it was shown that hookworm infection among school age children was 6.3%, *T*. *trichiura* prevalence was 1.1% and no *A*. *lumbricoides* infection was found [21]. A 2015 systematic review and geostatistical meta-analysis in sub-Saharan Africa showed 9.9% prevalence for hookworm, 0.4% prevalence for both *A*. *lumbricoides* and *T*. *trichiura* and 10.7% prevalence for overall STH in Burkina Faso, from 2000 onwards [22].

Since the early 2000s, deworming activities in Burkina Faso have been implemented through a number of different platforms as shown in Table 1. Firstly, the national lymphatic filariasis (LF) elimination program initiated annual mass drug administration (MDA) in 2001 with albendazole and ivermectin for LF elimination, targeting all individuals aged 5 years or older, and reached national coverage in 2005. Secondly, the national schistosomiasis and STH control program was established in 2004. Albendazole tablets were added to praziquantel distribution to treat school age children [23, 24]. The MDA was conducted once every two years in all 63 health districts reaching approximately 90% coverage in school age children [23]. In 2007, the national schistosomiasis and STH control program became part of the national integrated NTD program for the five major NTDs targeted by preventive chemotherapy [25]. Thirdly, deworming has also been implemented in pre-school age children (12–59 months old) together with vitamin A supplementation through Child Health Days in the country with support from HKI and UNICEF.

**Table 1:** Deworming activities through different platforms in Burkina Faso.

Population groups	Treatment drugs	Treatment frequency	Period	MDA platform	Notes
Pre-school age children (12–59 months)	Mebendazole	Twice a year	Since 2007	Vitamin A supplementation	Door-to-door distribution during Child Health Days that include vitamin A supplementation
School age children (5–14 years)	Albendazole	Once every two years	2004–2006	Schistosomiasis MDA (school- and community-based)	Albendazole treatment with praziquantel stopped after integration with LF MDA.
Total LF eligible population (≥5 years old)	Albendazole + ivermectin	Once a year (twice a year in 4 districts in Sud Ouest region)	Since 2001	LF MDA (community-based)	Geographical coverage was gradually scaled up to 100% in 2005, and then has been gradually scaled down since 2012.

With the LF MDA being the largest deworming program and being gradually stopped in more and more districts since 2012 in Burkina Faso after achieving the LF program objectives, it is essential to assess the STH situation in order to plan for the STH-specific treatment strategies in the post-LF MDA setting. In 2013, STH infections were assessed in school age children during a schistosomiasis sentinel site survey in 22 sentinel sites in 11 regions across the country. LF transmission assessment surveys (TAS) have been used to include STH assessment elsewhere through school-based surveys [26, 27]. In 2014, an assessment of STH infections during LF TAS was conducted in the Centre Nord region in Burkina Faso to test the feasibility of assessing STH through community-based LF TAS design, compared with the conventional school-based surveys. The current paper presents a full picture of current STH prevalence and distribution in the country, discusses the current STH situation and the future need for STH control in Burkina Faso, and demonstrates the feasibility of STH assessment during LF TAS at community level.

## Materials and Methods

### Ethical consideration

The survey was part of the monitoring and evaluation activities of the national integrated NTD program and was authorized by the Ethics Committee of the Ministry of Health of Burkina Faso. The surveys were conducted by the Ministry of Health monitoring and evaluation team. The populations were informed of the background of the surveys through the community health workers and town criers. Administrative and local authorities and community leaders were involved in the surveys. Parents were informed about the purpose and objectives of the survey through community meetings. They were also informed that they had the right to withdraw their children at any time of the survey. Verbal consent was given by parents for all children selected for the survey and recorded on survey forms and this was approved by the Ministry of Health as the literacy rate was low in rural areas in Burkina Faso. Written informed consent was obtained, before the survey started, from the head teachers of the schools as the legal guardian of all children in schools. Any children who did not want to participate were free to leave. Survey results were used for decision making for national strategy of STH control.

### Sentinel site surveys

At the beginning of the integrated national NTD program, 22 sentinel sites (schools) were purposefully selected in 2008 for schistosomiasis impact assessment based on prior knowledge for schistosomiasis. The 22 sites were located across 11 health regions (Boucle du Mouhoun, Cascades, Centre-Est, Centre Nord, Centre Ouest, Centre Sud, Est, Hauts Bassins, Nord, Sahel and Sud Ouest) with relatively even geographical distribution in the country [28]. Cross-sectional surveys in these sentinel sites for schistosomiasis were conducted in 2008 and 2013. At the same time, STH infections were also examined in selected school age children. Within each school, 16 boys and 16 girls from each of the 7–11 age groups (Classes 1–5), totalling approximately 160 children per school, were systemically selected and examined by parasitological examinations. If there were fewer children than required sample size in a school, additional children were selected from a neighbouring school within a five kilometer radius.

### Assessment through LF TAS

LF TAS have been used as a platform to assess the impact of MDA on STH in school-based surveys and to determine the treatment strategy for STH after community-wide LF MDA has been stopped [26, 27]. An STH assessment survey during LF TAS was conducted in 2014 in the Centre Nord evaluation unit (EU) to test the feasibility of STH assessment during community-based LF TAS survey. The survey design for LF TAS followed the WHO TAS guidelines [29] and the concurrent STH survey followed the then-draft and now-published WHO TAS-STH survey guidelines [30], assisted by the Survey Sample Builder tool developed by the Task Force for Global Health (http://www.ntdsupport.org/resources/transmission-assessment-survey-sample-builder). The Centre Nord EU consists of four health districts (implementation units). The EU, comprising a total of 1285 enumeration areas, had an estimated population of 1.5 million people and 60,120 children of 6–7 year-old in 2014. The primary school enrolment rate was 67%. Therefore, a community-based cluster survey was conducted in accordance with the WHO guidelines [29, 30]. In total, 42 clusters (villages) were selected and surveyed.

For the concurrent STH survey, a subset of 336 children of 6–7 years old was sampled in the same clusters (villages) as TAS. This gave rise to eight children per cluster. After the LF team selected the 6–7 year-old children for LF tests, additional STH technicians identified those children in the sample who would also be assessed for STH. In order to compare any differences with older age groups and also to facilitate the comparison with the conventional school-based STH survey, an additional group of 336 children aged 10–14 years old were sampled in the survey. These 10–14 year-old children were selected from the same households as the selected 6–7 year-old children, i.e. when one 6–7 year-old child was selected, one 10–14 year-old child from the same household was also selected. If there were more than one 10–14 year-old children in the household, a random selection was used. If there were no 10–14 year-old children in the household, the missing number of 10–14 year-old children was made up by random selection from other households with 6–7 year-old children being selected for LF tests.

The TAS-STH survey methodology uses the same critical cutoff values for decision-making as the standalone LF TAS [29, 30]. The critical cutoff values indicate the maximum number of positive cases that can be found in a given EU for clarifying the EU to a certain prescribed prevalence threshold, so that corresponding treatment strategies can be applied to the whole EU.

For comparison, a conventional school-based STH survey was conducted separately in the same EU according to the WHO recommendations [31]. Five primary schools were randomly selected in the EU. 50 children aged 10–14 years old from each school were selected and examined. If there were fewer than 50 children aged 10–14 years old in a school, additional children were selected from a neighbouring school within a five kilometer radius.

### Parasitological examinations

One stool sample was collected from each of the selected children in containers which were labeled with unique identification numbers. As described elsewhere [28], the samples were sent back to a local laboratory for examinations on the same day. The sample processing and examination methods were as described previously [32]. The Kato-Katz method was used to determine STH infections (hookworms, *A*. *lumbricoides* and *T*. *trichiura*). Two slides were prepared from each sample and examined on the same day. Eggs from each of these parasites were counted and individual egg counts were calculated and expressed as eggs per gram of faeces (epg).

### Data analysis

The data collected were entered into an Excel spreadsheet and double checked by biomedical technicians. The SPSS software (IBM, version 19) was used for statistical analysis. When calculating the overall prevalence in the country, the samples were weighted according to the proportion of the regional population among the total population and the Complex Samples module was used taking into consideration the cluster nature of school children using region as strata and school as clusters. The 95% confidence intervals (CIs) for prevalence were calculated using the CI calculator (available: http://vl.academicdirect.org/applied_statistics/binomial_distribution/ref/CIcalculator.xls). Arithmetic mean egg count from all subjects examined (including both positive and negative) was calculated. Individual egg count was categorized as light, moderate or heavy infection according to the WHO recommendations [33]. The Chi-squared test was used to compare differences in prevalence and the Kruskal-Wallis test was used to compare differences in mean egg counts. The dataset from the 2008 survey of the same 22 sentinel sites was not available for statistical comparison, therefore the STH prevalence data from the national survey report were used for descriptive comparison with the 2013 data [34]. For STH data from assessment during the LF TAS, the number of positive cases identified was used to clarify the prevalence threshold in the EU, against the pre-defined critical cut-off values in the WHO guidelines [30]. The coordinates of survey sites were collected using a handheld GPS device. Where there was an error, the location was estimated on the google map. The site location map was drawn in ArcMap version 10 (ESRI, Redlands, CA).

## Results

### Sentinel site survey

Table 2 summarizes the 2013 survey results from the 22 sentinel sites. In total, 3,514 school age children (1,748 boys and 1,766 girls) were examined. Overall STH prevalence in children tested was very low at 1.3% (95% CI: 1.0–1.8%), ranging from 0% to 6.9% among 22 sites (median 0.6%). Among three major STH species, hookworm was the main species detected. *A*. *lumbricoides* and *T*. *trichiura* were only detected in four and two cases respectively, and therefore prevalence and mean egg counts of these two parasites were not calculated separately. As in Table 2, overall hookworm prevalence was 1.2% (95% CI: 0.9–1.6%), ranging from 0% to 6.3% (median 0.6%). All individual hookworm infections were light infections with mean egg count of 2.1 epg (95% CI: 0–4.2 epg).

**Table 2:** Results of STH infections in school age children from the 22 sentinel sites in Burkina Faso in 2013.

Regions	No of children examined	Hookworm		Overall STH
		Prevalence (%) (95% CI)	Mean egg count (epg)	Prevalence (%) (95% CI)
Boucle du Mouhoun	429	0.6 (0.2–2.3)	0.3 (0–0.7)	0.6 (0.2–2.3)
Cascades	169	1.9 (0.6–5.4)	0.6 (0–1.3)	1.9 (0.6–5.4)
Centre Est	343	1.9 (0.9–4.0)	1.0 (0.2–1.8)	2.8 (1.5–5.3)
Centre Nord	362	0	0	0
Centre Ouest	355	3.4 (1.9–6.1)	14.9 (3.3–26.6)	3.8 (2.2–6.4)
Centre Sud	190	0.3 (0.1–1.8)	0.1 (0–0.2)	0.6 (0.2–2.3)
Est	375	1.3 (0.5–3.2)	0.8 (0–1.7)	1.6 (0.7–3.7)
Hauts Bassins	454	2.1 (1.1–3.8)	2.2 (0–4.5)	2.1 (1.1–3.8)
Nord	353	0.3 (0.1–1.8)	0.1 (0–0.2)	0.3 (0.1–1.8)
Sahel	296	0	0	0
Sud Ouest	187	0	0	0
**Total**	**3514**	**1.2 (0.9–1.6)**	**2.1 (0–4.2)**	**1.3 (1.0–1.8)**

**Fig 1:**
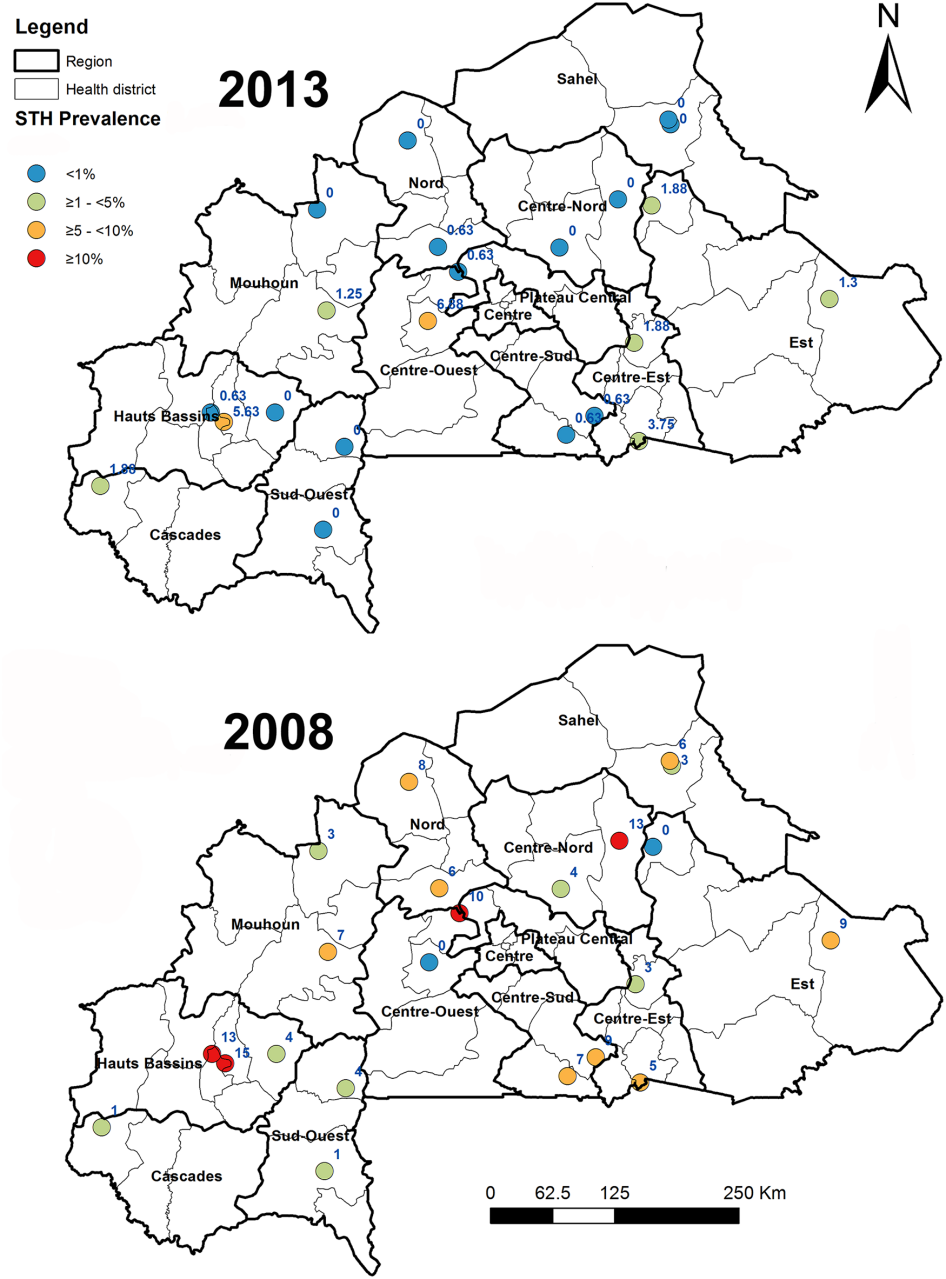
Prevalence of STH infections among school age children in 22 schistosomiasis sentinel sites, Burkina Faso from sentinel site surveys in 2008 and 2013.

The prevalence at each of the 22 sentinel sites in 2013 and the prevalence at each site in 2008 from these same sites taken from the national survey report are shown in Fig 1. It showed a general reduction in the STH prevalence in 2013 compared with the STH prevalence in 2008 across the 22 sentinel sites in the country. But statistical comparison was not possible due to the lack of availability of the 2008 dataset.

There were significant differences in hookworm prevalence between regions (Chi-square test, χ^2^ = 36.447, *P*<0.001). Centre Ouest region had the highest prevalence of 3.4% (95% CI: 1.9–6.1%) and mean egg count of 14.9 epg (95% CI: 3.3–26.6 epg), followed by Hauts Bassins region of 2.1% (95% CI: 1.1–3.8%) and 2.2 epg (95% CI: 0–4.5 epg) respectively (Table 2). There was no significant difference in hookworm infection between boys, with prevalence of 1.0% (95% CI: 0.6–1.6%) and mean egg count of 1.0 epg (95% CI: 0.2–1.8 epg), and girls, with prevalence of 1.3% (95% CI: 0.9–2.0%) and mean egg count of 3.1 epg (95% CI: 0–7.3 epg), (Chi-square test for prevalence, χ^2^ = 0.695, *P*>0.05; Kruskal Wallis test for mean egg count, H = 0.409, *P*>0.05).

### Assessment through LF TAS

**Fig 2:**
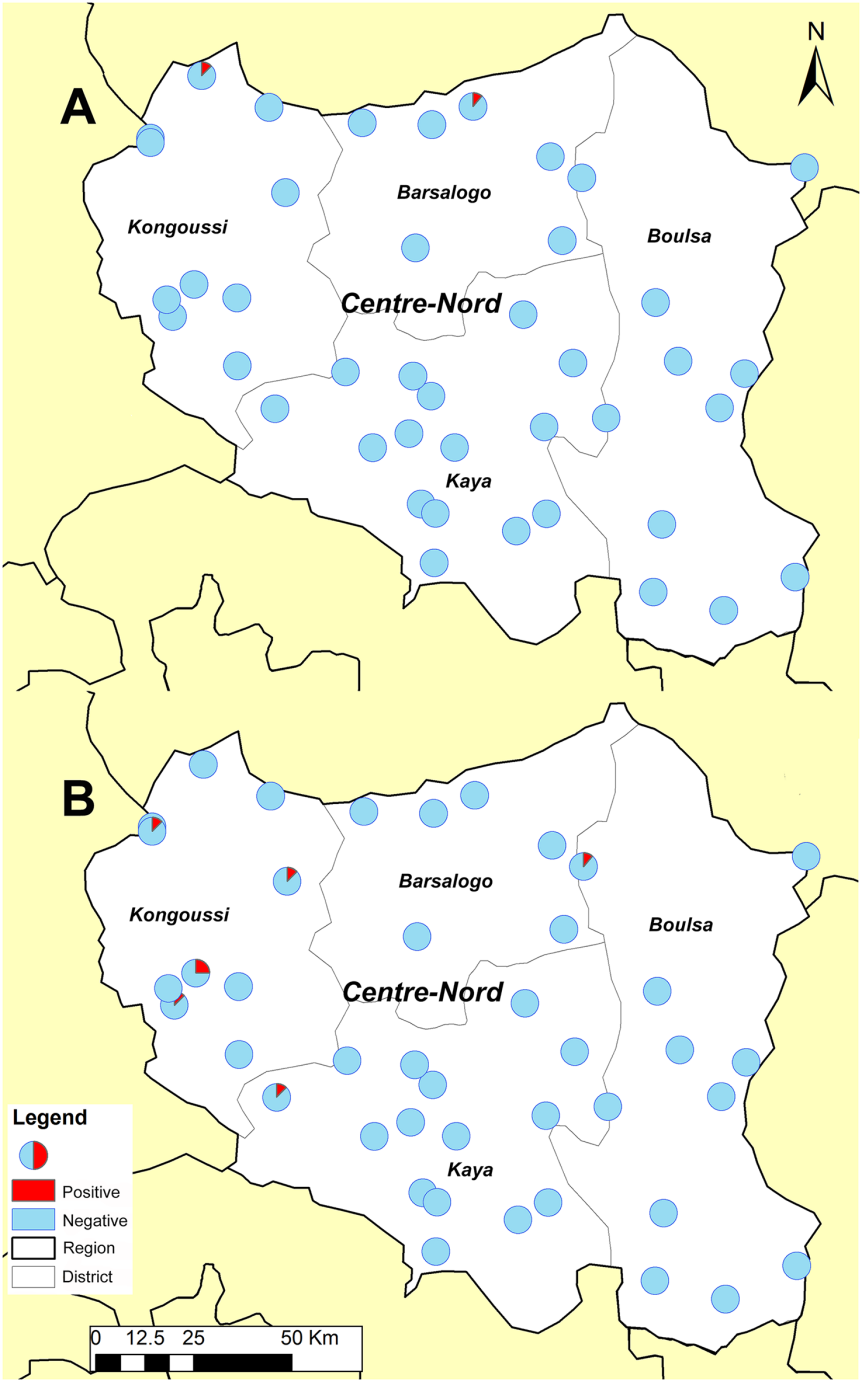
Distribution of positive STH cases in surveyed clusters in the Centre Nord EU from the LF TAS-STH assessment in 2014. A) 6–7 years old, B) 10–14 years old.

Table 3 summarizes the results of STH assessment during the LF TAS in 2014 (LF results are not presented in this paper). In total, 351 children (184 boys and 167 girls) aged 6–7 years old were examined for STH infection during the TAS in the EU. Two cases of STH infection were identified with estimated prevalence of 0.6% (95% CI: 0.2–2.1%) in children tested. Similarly, 345 children (164 boys and 181 girls) aged 10–14 years old were examined for STH infection during the TAS, and seven cases of STH infection was identified with estimated prevalence of 2.0% (95% CI: 1.0–4.1%) in children tested. All identified positive cases were hookworm infections and no infection with *A*. *lumbricoides* or *T*. *trichiura* was found. In both age groups, there was no difference in STH infections between boys and girls (Chi-square test, χ_6−7_^2^ = 1.826, χ_10−14_^2^ = 0.264, *P*>0.05). Although there were more STH cases identified in the 10–14 year-old group, the difference was not statistically significant (Chi-square test, χ^2^ = 2.902, *P>*0.05). Both age groups categorized the EU to be within 2% to <10% in STH prevalence according to the threshold cut-off values (Table 3). The general distribution of the positive cases in the EU is shown in Fig 2. The positive cases were aggregated in the north part of the region, particularly in the Kongoussi health district.

**Table 3:** Results of STH assessment during LF TAS and school-based survey in Centre Nord in Burkina Faso in 2014.

Survey group	No of children examined	No of children positive	Estimated prevalence (%) (95% CI)	Critical cut-off value for <10% prevalence for cluster sampling [30]	STH prevalence according to the cut-off value
6–7 years old	351	2	0.6 (0.2–2.1)	1–20	2% to <10%
10–14 years old	345	7	2.0 (1.0–4.1)	1–20	2% to <10%
10–14 years old (school-based)	250	0	0 (0–1.5)	-	-

In the conventional school-based survey, a total of 250 school children aged 10–14 years (124 boys and 126 girls) were examined, and no STH infection was identified. The estimated STH prevalence was 0% (95% CI: 0–1.5%). When compared with the two TAS survey groups, there was no significant difference with the 6–7 year-old group (Chi-square test, χ^2^ = 1.429, *P*>0.05), but there was a significant difference with the 10–14 year-old group (Chi-square test, χ^2^ = 5.133, *P<*0.05).

## Discussion

Survey of the 22 sentinel sites across 11 regions showed that STH infections in school age children in Burkina Faso were at a low level in the majority of the country’s endemic districts. The residual infections were mainly hookworm infections, while *Ascaris* and *Trichuris* infections were very rarely seen. No moderately or heavily infected cases were found during the survey. This is in line with the TAS-STH results in the Centre Nord region. It is suggested that over a decade of large-scale preventive chemotherapy targeting different age groups through various program platforms implemented in Burkina Faso has effectively controlled STH in the country.

Burkina Faso was one of the first countries in sub-Saharan Africa to start national NTD programs with large-scale preventive chemotherapy with external financial and technical support. The national schistosomiasis and STH program established in 2004 was the first national STH program in the country. The results from the schistosomiasis baseline survey at the time showed that STH, particularly hookworm, was endemic in Burkina Faso, but with a relatively low prevalence [21]. Regardless of the low prevalence, the national program decided to conduct the large scale MDA intervention together with the schistosomiasis MDA. This decision was based on: 1) Burkina Faso was and still is among the poorest countries in the world according to the Human Development Index [35]; 2) there was high prevalence of anemia in the country [36, 37], and hookworm infection is a risk factor for anemia in women and children [3, 37–39]; 3) adding albendazole to praziquantel distribution to treat school age children does not incur extra cost for drug delivery; and 4) deworming is among the most cost-effective investments in global health and benefits of deworming were demonstrated [39–41].

With all deworming activities through community-wide LF MDA, school-based and community-based schistosomiasis and STH MDA, and Child Health Days, the program rapidly achieved national coverage with all endemic health districts targeted, with good treatment coverage in pre-school age children and school age children. Burkina Faso successfully achieved and maintained the target of at least 75% of national coverage as recommended by WHO [11, 15]. The current results of sentinel site survey suggest that STH have been successfully controlled as a public health problem in school age children in Burkina Faso as STH infection of moderate or high intensity from the surveys was below the threshold of 1% as defined by WHO [11]. Integrated, community-wide MDA programs for schistosomiasis and STH can be highly cost effective, even in communities with low disease burden in any helminth group [42]. Multi rounds of large scale community-wide LF MDA may have helped to achieve successful control of STH in Burkina Faso. However, lack of baseline data prior to the commencement of LF MDA for statistical comparison makes it difficult to attribute conclusively the low STH prevalence to the impact of such MDA activities.

Despite the achievements in Burkina Faso as shown by the data, it is however noted that there are still some hot spot infections as there were three sentinel sites showing hookworm prevalence being 6.9%, 5.6% and 3.8% respectively from the survey, particularly with one site in Centre Ouest showing increased prevalence from 2008 (Fig 1). Single dose of albendazole has 87.8% cure rate and >90% fecal egg count reduction rate for hookworm [43], and annual community-wide mass treatment such as LF MDA is expected to reduce hookworm infection to ground level within a few years [44]. The fact that such hot spots for hookworm infection still existed after many years of large scale MDA intervention suggests that some focal factors may have affected the impact of the treatment. While research is needed on possible local factors that contributed to the persistence or increase of hookworm infection in these locations, potential reasons may include: 1) poor focal coverage–the overall national treatment coverage may have been high, but treatment coverage at some communities may not have been satisfactory, and 2) there may be some particular local factors, such as lack of clean water, poor hygiene and sanitation [7]. The national program needs to pay special attention to such hot spot communities, i.e. providing supervision and monitoring in future MDAs to ensure the high treatment coverage.

There are other limitations in this study. Firstly, the sentinel sites were selected for schistosomiasis impact assessment according to the endemicity of schistosomiasis. Therefore the results from these sites may not represent the true STH situation across all the communities in the country. Secondly, the survey was conducted among school age children attending schools. Given the low school enrolment rate in Burkina Faso, a third of school age children were in communities who were not subject to sampling and who may be more disadvantaged and prone to STH infection. The current results from the community-based STH assessment during LF TAS versus conventional school-based survey may reflect this. On the other hand, hookworm is the main STH species in Burkina Faso and adult population harbors significant worm load [44–46]. The survey results in school age children may represent better the *Ascaris* and *Trichuris* situation, but may not represent the hookworm situation in the whole communities. Taken together, it is advisable that the national program should consider community-based assessment and include survey in adult population to confirm the current STH, particularly hookworm situation in the country.

LF MDA with albendazole and ivermectin is the largest community-wide deworming activity in Burkina Faso, and this has been stopped in more and more districts and is expected to stop in all districts soon due to meeting stopping LF MDA criteria. To consolidate the impact already achieved and to avoid recrudescence, the national program should continue to implement STH treatment through 1) LF MDA where LF MDA has not been stopped; 2) adding albendazole to schistosomiasis MDA where LF MDA has been stopped; and 3) Child Health Days. Given the low level of STH infection from the current results, the country may be in a good position to pursue interruption of STH transmission [44]. Research is needed on what strategies are required to sustain the gain and to interrupt STH transmission in such settings as in Burkina Faso, particularly after community-wide LF MDA has stopped. Where hookworm is the dominant STH species, mass treatment of all age groups is recommended [44]. Bearing in mind that chemotherapy alone may not be enough to interrupt STH transmission [47], other strategies would be needed, i.e. provision of clean water, hygiene and sanitation. Funding should be sourced to conduct such research for cost-effective strategies in Burkina Faso to interrupt STH transmission.

LF TAS provides a perfect timing and platform to assess the STH situation to determine the STH-specific deworming strategy after LF MDA is stopped. In the current study, a community-based cluster survey was tested in one EU (Centre Nord region), and we assessed the feasibility of integrating STH assessment with community-based cluster survey for LF TAS. The process of the survey suggests that the joint assessment in community-based surveys is indeed feasible and efficient, testing either 6–7 years old (same as LF TAS target) or 10–14 years old. Sampling 10–14 year-olds detected more STH cases, but the result was not significantly different from that of sampling 6–7 years old. The results from both groups classified the EU as between 2% to <10% STH prevalence. The TAS design for STH assessment assumed a design effect of 2.0 for the cluster sampling. In our survey, the actual design effect was 0.943 for 6–7 years old group and 1.165 for 10–14 years old group. Therefore the survey was sufficiently powered for estimating the STH situation.

Comparing with the conventional school-based surveys, community-based testing of 10–14 years old using the LF TAS platform did show significantly higher STH prevalence. It is noted that the separate conventional school-based survey had to be conducted shortly after MDA that had not allowed sufficient time for re-infection. Therefore, the results of the conventional school-based survey may represent an underestimate of the true prevalence in school age children and account for the difference with the TAS-STH results. However, considering the results from two sentinel sites in the same region surveyed in 2013 with larger sample size per school that did not detect any STH infection either (Fig 1), the results from the conventional school-based survey may have been a true representation of prevalence using such a survey methodology. This suggests that community-based TAS-STH may give a better estimate of STH situation in low prevalence areas with low school enrollment rate such as in Burkina Faso. In particular, the TAS-STH results provide a clear indication of geographical aggregation of clusters with STH infections. This provides national program managers a powerful tool for program decision. Although conventional school-based survey is easier to organize, LF TAS-based assessment provides better estimate of STH situation and is integrated with the LF TAS, therefore the national program will continue to conduct such assessments in other regions.

In conclusion, through large-scale preventive chemotherapy Burkina Faso have successfully controlled STH in school age children in the country. Research is needed on potential reasons and factors that hookworm infection persists in some locations after many rounds of MDA and for future strategies to consolidate the gains made from interventions to date and to target interruption of STH transmission in Burkina Faso. LF TAS provides one feasible and efficient platform to assess the STH situation for post LF MDA decision making and should be further examined and implemented as a monitoring and evaluation tool.

## Supporting Information

S1 Checklist STROBE Checklist. (DOC)
