# Supplementary material for: Successful Control of Soil-Transmitted Helminthiasis in School Age Children in Burkina Faso and an Example of Community-Based Assessment via Lymphatic Filariasis Transmission Assessment Survey
Source: PLoS Negl Trop Dis. 2016 May 10;10(5):e0004707. doi: 10.1371/journal.pntd.0004707 (PMC4862685; doi:10.1371/journal.pntd.0004707)
Supplement: S1 Checklist — (DOC) [file pntd.0004707.s001.doc]

STROBE Statement—Checklist of items that should be included in reports of ***cross-sectional studies***

|  | Item No | Recommendation |
| --- | --- | --- |
| **Title and abstract** | 1 | (*a*) Indicate the study’s design with a commonly used term in the title or the abstract Yes, pages #1-2 |
|  |  | (*b*) Provide in the abstract an informative and balanced summary of what was done and what was found Yes, page #2 |
| Introduction | | |
| Background/rationale | 2 | Explain the scientific background and rationale for the investigation being reported Yes, pages #5-8 |
| Objectives | 3 | State specific objectives, including any prespecified hypotheses Yes, pages #7-8 |
| Methods | | |
| Study design | 4 | Present key elements of study design early in the paper Yes, pages #9-10 |
| Setting | 5 | Describe the setting, locations, and relevant dates, including periods of recruitment, exposure, follow-up, and data collection Yes, pages #9-11 |
| Participants | 6 | (*a*) Give the eligibility criteria, and the sources and methods of selection of participants Yes, pages #9-11 |
| Variables | 7 | Clearly define all outcomes, exposures, predictors, potential confounders, and effect modifiers. Give diagnostic criteria, if applicable Yes, pages #11-12 |
| Data sources/ measurement | 8* | For each variable of interest, give sources of data and details of methods of assessment (measurement). Describe comparability of assessment methods if there is more than one group Yes, pages #11-12 |
| Bias | 9 | Describe any efforts to address potential sources of bias Yes, pages #11-12 |
| Study size | 10 | Explain how the study size was arrived at Yes, pages #9-10 |
| Quantitative variables | 11 | Explain how quantitative variables were handled in the analyses. If applicable, describe which groupings were chosen and why N/A |
| Statistical methods | 12 | (*a*) Describe all statistical methods, including those used to control for confounding Yes, pages #11-12 |
|  |  | (*b*) Describe any methods used to examine subgroups and interactions Yes, page #12 |
|  |  | (*c*) Explain how missing data were addressed N/A |
|  |  | (*d*) If applicable, describe analytical methods taking account of sampling strategy Yes, page#11 |
|  |  | (*e*) Describe any sensitivity analyses N/A |
| Results | | |
| Participants | 13* | (a) Report numbers of individuals at each stage of study—eg numbers potentially eligible, examined for eligibility, confirmed eligible, included in the study, completing follow-up, and analysed Yes, pages #12-15 |
|  |  | (b) Give reasons for non-participation at each stage N/A |
|  |  | (c) Consider use of a flow diagram N/A |
| Descriptive data | 14* | (a) Give characteristics of study participants (eg demographic, clinical, social) and information on exposures and potential confounders Yes, pages #12-15 |
|  |  | (b) Indicate number of participants with missing data for each variable of interest N/A |
| Outcome data | 15* | Report numbers of outcome events or summary measures Yes, pages #12-15 |
| Main results | 16 | (*a*) Give unadjusted estimates and, if applicable, confounder-adjusted estimates and their precision (eg, 95% confidence interval). Make clear which confounders were adjusted for and why they were included Yes, pages #12-14 |
|  |  | (*b*) Report category boundaries when continuous variables were categorized N/A |
|  |  | (*c*) If relevant, consider translating estimates of relative risk into absolute risk for a meaningful time period N/A |
| Other analyses | 17 | Report other analyses done—eg analyses of subgroups and interactions, and sensitivity analyses N/A |
| Discussion | | |
| Key results | 18 | Summarise key results with reference to study objectives Yes, page #16 |
| Limitations | 19 | Discuss limitations of the study, taking into account sources of potential bias or imprecision. Discuss both direction and magnitude of any potential bias Yes, pages #17-18 |
| Interpretation | 20 | Give a cautious overall interpretation of results considering objectives, limitations, multiplicity of analyses, results from similar studies, and other relevant evidence Yes, pages #18-19 |
| Generalisability | 21 | Discuss the generalisability (external validity) of the study results N/A |
| Other information | | |
| Funding | 22 | Give the source of funding and the role of the funders for the present study and, if applicable, for the original study on which the present article is based Yes, given in online submission forms |

*Give information separately for exposed and unexposed groups.

**Note:** An Explanation and Elaboration article discusses each checklist item and gives methodological background and published examples of transparent reporting. The STROBE checklist is best used in conjunction with this article (freely available on the Web sites of PLoS Medicine at http://www.plosmedicine.org/, Annals of Internal Medicine at http://www.annals.org/, and Epidemiology at http://www.epidem.com/). Information on the STROBE Initiative is available at www.strobe-statement.org.
